# Early-onset colorectal cancer: a retrospective study of demographic, clinicopathological, and molecular characteristics in a single Chinese center

**DOI:** 10.1186/s40001-025-02819-6

**Published:** 2025-08-02

**Authors:** Zongyao Chen, Linhan Ye, Yuhang Liu, Menghang Geng, Shuangya Deng, Weidong Chen

**Affiliations:** 1 Department of Surgery, TUM University Hospital, Klinikum Rechts Der Isar, Munich, Germany; 2 Department of General Surgery, Central Hospital of Shaoyang, Shaoyang, Hunan Province People’s Republic of China; 3 Guilin Hospital of the Second Xiangya Hospital, Central South University, Guilin, People’s Republic of China; 4 Department of General Surgery, The Second Xiangya Hospital, Central South University, Changsha, 410011 Hunan Province People’s Republic of China

**Keywords:** Colorectal cancer, Early-onset, Epidemiology, Mismatch repair deficiency, Tumor location

## Abstract

**Background:**

Early-onset CRC is typically defined as CRC diagnosed in individuals under the age of 50 years. The global incidence and mortality rates of early-onset CRC have gradually increased. The clinicopathological features and pathogenesis of early-onset CRC have still not been fully elucidated, and related data are lacking in China. This research aimed to examine the demographic and clinicopathological characteristics of early-onset CRC patients in China.

**Materials and methods:**

This retrospective study included all patients newly diagnosed with CRC between 2019 and 2021 in the General Surgery Department of the Second Xiangya Hospital, Central South University.

**Results:**

A total of 1206 CRC cases were included. Among them, 180 cases (14.9%) were early-onset CRC, and 1026 cases were late-onset CRC, all of which were collected and analyzed. Early-onset CRC patients had significantly longer median symptom durations (90 vs. 60 days, *P* < 0.001). Patients with late-onset CRC less commonly had a family history than patients with early-onset CRC did (25.60% vs. 17.93%, *P* = 0.022). There was no direct relationship between symptom duration and disease stage at presentation in early-onset CRC patients (*P* = 0.750). Early-onset CRC patients were more likely to present with advanced disease (stage IV) compared to late-onset CRC patients (24.44% vs. 13.45%, *P* < 0.001). Additionally, early-onset CRC patients were more likely to present with poorly differentiated tumors (29.81% vs. 12.70%, *P* < 0.001) and with mucinous or signet-ring cell histology (22.40% vs. 14.17%, *P* = 0.011) compared to late-onset CRC patients. Deficient mismatch repair (dMMR) tumors were more common in early-onset CRC patients (15.90% vs. 6.28%, *P* < 0.001). Among early-onset CRC patients, no significant differences were observed in age, sex, BMI, or tumor pathology between those with right-sided and left-sided tumors.

**Conclusions:**

Early-onset CRC has different epidemiology, pathology, and molecular features than late-onset CRC in China. More research is needed to better understand the pathophysiology of early-onset CRC and why there are different characteristics between the two types of CRC.

## Introduction

CRC is the third most common malignancy and the second leading cause of cancer mortality globally, and patients who die from CRC account for 9.4% of all cancer-related fatalities [1]. The overall incidence of CRC has shown either a stable or declining trend, primarily due to the popularization of colonoscopy screening [2]. However, the incidence and mortality rates of CRC in patients younger than 50 years (early-onset CRC) have gradually increased worldwide [3–6]. Early-onset CRC is the second most common form of malignancy in this age group and the third leading cause of cancer-related death in the United States, with at least one in ten newly diagnosed CRC patients being diagnosed with early-onset CRC [7, 8]. The incidence of early-onset CRC varies across different regions globally. In the United States, between 2000 and 2013, the incidence of early-onset CRC increased by 22% [9]. In Europe, from 2004 to 2016, the average annual percentage change in the incidence of early-onset CRC in patients aged 20–29 years was 7.9%, 4.9% for patients aged 30–39 years, and 1.6% for those aged 40–49 years [10].

Despite the global trend of an aging population, by 2030, approximately 11% of colon cancers and 23% of rectal cancers will occur among people under the age of 50 years [11]. It is unknown why the incidence and mortality of early-onset CRC are increasing. There has previously been evidence that specific early life exposures, such as a Western diet [12, 13], increased stress [14, 15], excessive antibiotic use [16, 17], altered gut microbiome [18–21], smoking [22], obesity [23–26] and sedentary lifestyle [27], are associated with an increased risk of developing early-onset CRC. Mounting evidence indicates that nutrition, numerous drugs, and environmental factors may all contribute to the cellular epigenetic changes that result in certain tumor molecular subtypes [28, 29]. Previous studies have shown that early-onset CRC patients have different features from late-onset CRC patients in that the former tend to present with advanced disease and often exhibit poor histopathological features [30–35]. The distal colon and rectum are the most common locations for early-onset CRC tumors [9, 31, 36].

As the incidence of early-onset CRC continues to rise, a clearer understanding of its clinical and pathological features is urgently needed. This study was conducted to characterize the demographic, clinicopathological, and molecular features of early-onset CRC in a Chinese population and to compare them with those of late-onset CRC. We hypothesized that early-onset CRC exhibits distinct clinicopathological and molecular characteristics compared to late-onset CRC, which may help improve understanding of its underlying biology and inform diagnostic strategies.

## Materials and methods

**Fig. 1:**
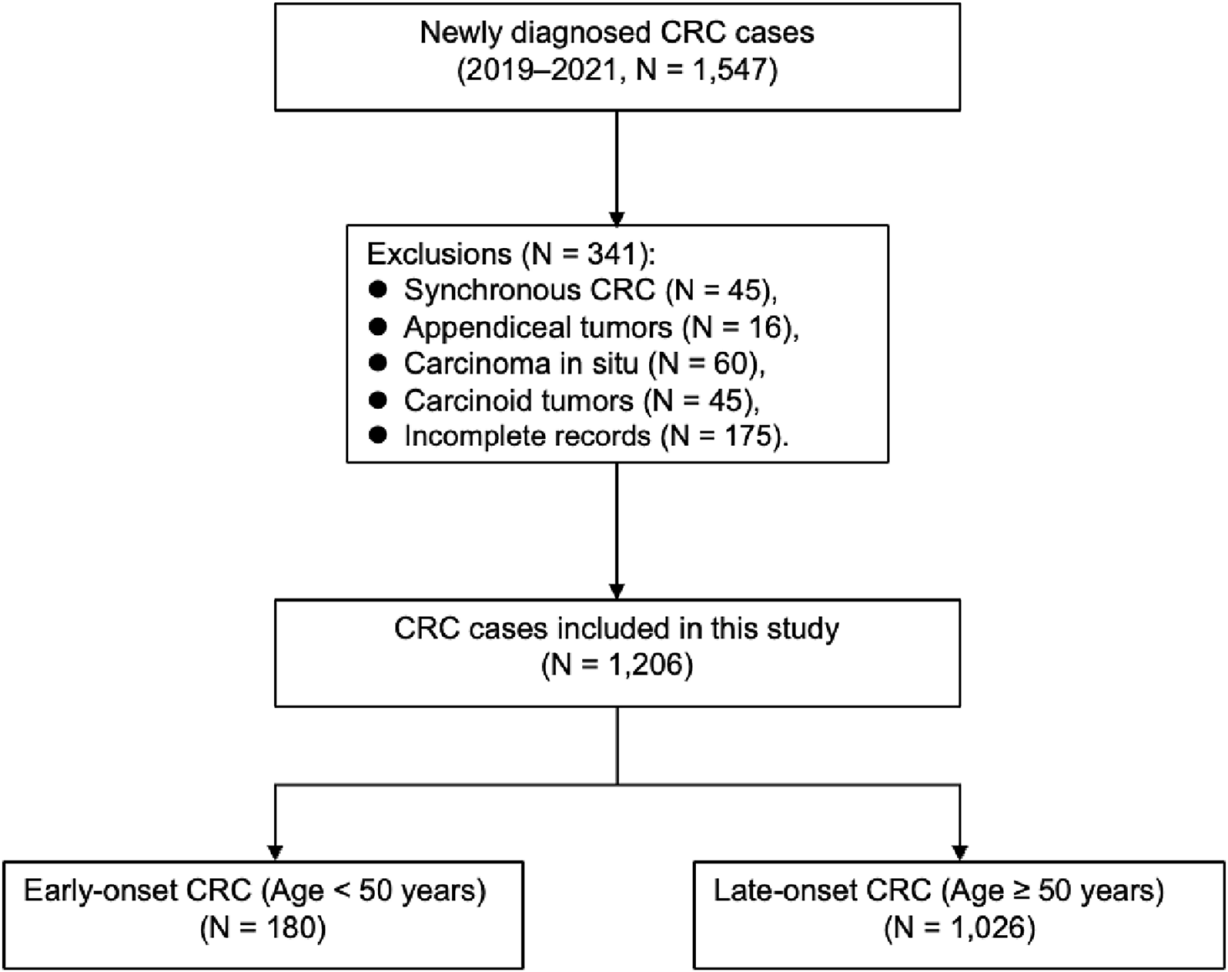
Flowchart of patient selection and classification in the study cohort

*Patients and information collection* We included patients who were newly diagnosed with CRC at the General Surgery Department of the Second Xiangya Hospital, Central South University, from 2019 to 2021. We excluded patients with synchronous CRC, appendiceal tumors, carcinoma in situ, carcinoid tumors, or incomplete records (Fig. 1).

We collected the following information for each patient: (1) demographic and clinical characteristics, including sex, age at diagnosis, body mass index (BMI), tumor location, presenting symptoms, family history of cancer, and treatment modality; (2) duration of symptoms prior to diagnosis; (3) tumor staging, evaluated according to the 8th edition of the American Joint Committee on Cancer (AJCC) staging system. Pathological staging was used for patients who underwent surgical resection, while clinical staging was applied for non-surgical cases based on imaging and multidisciplinary assessment; (4) histopathological features, including tumor histology, degree of differentiation, and the presence of perineural and/or vascular invasion; (5) microsatellite instability (MSI) status, determined by immunohistochemical expression of mismatch repair (MMR) proteins; and (6) survival-related data, including survival status, year of diagnosis, year of follow-up, year of death (if applicable), and overall survival (OS) in months.

(1) Early-onset CRC was defined as being diagnosed before the age of 50, because this is when most national screening programs begin (3–5). (2) Body mass index (BMI) (calculated as weight in kilograms divided by height in meters squared) was based on the height and weight of patients who started clinical symptoms. We defined underweight as a BMI < 18.5, normal weight as a BMI of 18.5–24.9, overweight as a BMI of 25.0–29.9, and obesity as a BMI ≥ 30.0. (3) Clinical symptoms were defined as intestinal bleeding, abdominal pain, changes in bowel habits (number of daily stool changes, constipation, diarrhea), bowel obstruction, other symptoms (anemia, imaging abnormalities, abdominal mass, abdominal distension, etc.), and colonoscopy screening. For patients with multiple initial symptoms, we selected the chief presenting symptom as the symptom that prompted the diagnosis. (4) Symptom duration was calculated based on the patient's self-reported duration of the primary presenting symptom at the time of the first visit. The symptom duration was set to zero days for individuals discovered by chance. (5) Right-sided colon tumors were defined as tumors arising at the cecum, ascending colon, and hepatic flexure colon. Left-sided colon tumors were defined as tumors arising at the splenic flexure, descending colon, and sigmoid colon. Left-sided CRC tumors included left-sided colon tumors and rectal tumors. (6) We defined MMR deficiency (dMMR) tumors as those lacking expression of at least one mismatch repair (MMR) protein (MLH1, MSH2, MSH6 or PMS2), as shown by immunohistochemistry.

*Patient and public involvement* This study was a retrospective study based on the abstraction of medical records. The study does not include patient advisors, as patients were not involved in the recruitment or conduct of the study. The study findings will not be disseminated directly to patients, although the findings will inform quality improvement initiatives in hospitals after the dissemination of the study results.

### Statistical analysis

SPSS was used to handle the data and perform the statistical analysis (version 22.0; SPSS Inc., Chicago, IL, USA). The means and standard deviations were computed from the measurement data, and the mean values were compared via an independent sample *t* test or variance analysis. We calculated medians and interquartile ranges (IQRs) for symptom duration and compared distributions by age group via the Wilcoxon rank-sum test. Pearson’s 2 test or Fisher’s exact probability approach was used to determine the frequency and percentage of qualitative data. *P* < 0.05 was used to determine statistical significance (two-tailed test).

## Results

*Demographic characteristics* A total of 1206 CRC cases were included. All 180 early-onset CRC patients (< 50 years), accounting for 14.9% of the cohort, were included. The remaining 1026 late-onset CRC patients (≥ 50 years) were also fully included for comparative analysis (Fig. 1). Patient demographics are summarized in Table 1. The median age at diagnosis was 41.81 ± 7.2 years for early-onset CRC patients and 62.13 ± 8.59 years for late-onset CRC patients. In terms of sex, 51.70% of the patients with early-onset CRC were female, whereas 34.20% of the patients with late-onset CRC were female (*P* < 0.001; Table 1). Compared with early-onset CRC patients, late-onset CRC patients had a greater BMI (mean: 23.80 ± 3.04 vs. 22.84 ± 3.02 kg/m^2^); moreover, late-onset CRC patients were more likely to be overweight (29.82% vs. 19.40%, *P* = 0.006; Table 1). Patients with late-onset CRC less commonly had a family history than patients with early-onset CRC did (25.6% vs. 17.93%, *P* = 0.022; Table 1). This study revealed no differences in the percentage of patients who received or did not receive chemotherapy for early and late-onset CRC patients (41.7% vs. 38.89%, *P* = 0.535; Table 1).

**Table 1:** Baseline clinical characteristics of EOCRC and LOCRC patients

	Early-onset CRC (< 50 years, *N* = 180)		Late-onset CRC (≥ 50 years, *N* = 1026)		
Average age	41.81 ± 7.2		62.13 ± 8.59		
	*N*	%	*N*	%	
Sex					< 0.001
Female	93	51.70%	351	34.20%	
Male	87	48.30%	675	65.80%	
Tumor location					0.901
Right-sided colon	22	12.22%	128	12.48%	
Transverse colon	10	5.56%	67	6.53%	
Lift-sided colon	61	33.89%	363	35.38%	
Rectum	87	48.33%	468	45.61%	
Clinical symptoms					
Intestinal bleeding	81	45.00%	487	47.47%	0.596
Abdominal pain	47	26.10%	217	21.15%	0.165
Change in bowel habits	43	23.90%	181	17.64%	0.060
Bowel obstruction	0	0.00%	32	3.12%	0.032
Others	4	2.20%	15	1.46%	0.666
Colonoscopy screen	5	2.80%	94	9.16%	0.006
Body Mass Index (kg/m^2^)					
Mean (std)	22.84 ± 3.02		23.80 ± 3.04		
Underweight [< 18.5]	14	7.80%	63	6.14%	0.504
Normal weight [18.5–24.9]	127	70.60%	636	61.99%	0.031
Overweight [25.0–29.9]	35	19.40%	306	29.82%	0.006
Obese [≥ 30.0]	4	2.20%	21	2.05%	1
Family history of CRC					0.022
Yes	46	25.60%	184	17.93%	
No	134	74.40%	842	82.07%	
Symptoms duration, days					< 0.001*
Median (IQR)	90 (30–180)		60 (20–150)		
Mean (SD)	164.1 ± 213.1		31.0 ± 111.3		
Surgery					0.147
Yes	161	89.44%	953	92.88%	
None	19	10.56%	73	7.12%	
Received chemotherapy					0.535
Yes	75	41.70%	399	38.89%	
None	105	58.30%	627	61.11%	
Survival (OS, months)					0.128
Median	52.0 (42.0–62.0)		50.0 (42.0–59.8)		
Mean (SD)	50.9 ± 14.9		48.6 ± 16.1		

CRC colorectal cancer, EOCRC early-onset colorectal cancer, LOCRC late-onset colorectal cancer

*Analysis by *T* test

*Symptoms and symptom duration* There were no significant differences in presenting symptoms between the two age groups; intestinal bleeding was the most common chief presenting symptom in both age groups. Approximately 2.80% of early-onset CRC patients were detected via colonoscopy, whereas approximately 9.16% of late-onset CRC patients were detected via colonoscopy (*P* = 0.006; Table 1). Compared with that in late-onset CRC patients, the median symptom duration was significantly longer in early-onset CRC patients (90 days [IQR 30–180] vs. 60 days [IQR 20–150], *P* < 0.001; Table 1), and the mean symptom duration was likewise longer in younger patients (164.1 ± 213.1 vs. 31.0 ± 111.3, *P* < 0.001; Table 1). There was no direct relationship between symptom duration and disease stage at presentation in early-onset CRC patients (median days, 90 [IQR 30–180] vs. 90 [IQR 60–180]; mean days, 167.1 ± 236.6 vs. 156.3 ± 164.0, *P* = 0.750; Table 2).

**Table 2:** Symptoms duration in EOCRC patients with different tumor stages

	Stages III–IV	Stages I–II	*P*
Symptoms duration, days			
Median (IQR)	90 (30–180)	90 (60–180)	0.750*
Mean (SD)	167.1 ± 236.6	156.3 ± 164.0	

*Analysis by *T* test

**Fig. 2:**
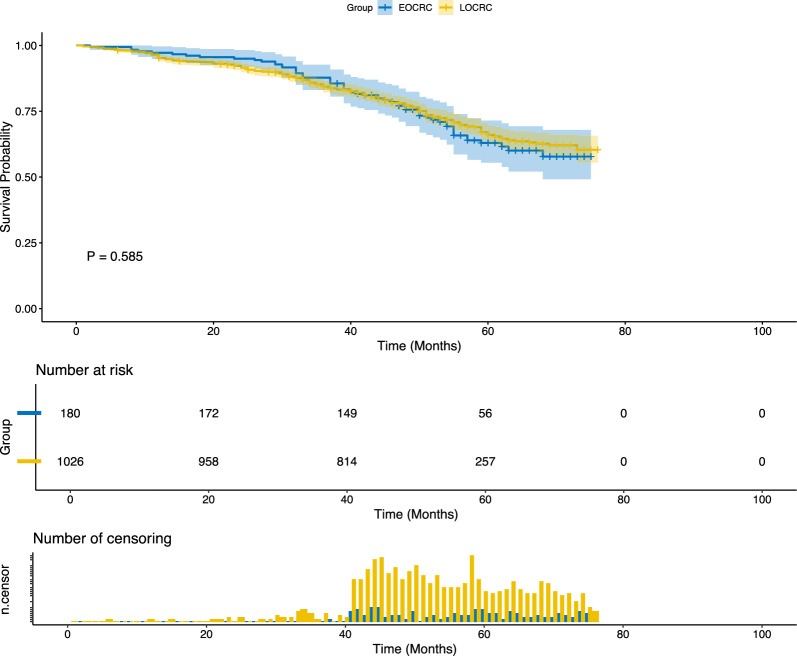
Survival analysis of EOCRC and LOCRC patients

*Survival analysis* A total of 1206 patients were included in the survival analysis, with 180 early-onset CRC and 1026 late-onset CRC cases. Kaplan–Meier curves showed no significant difference in overall survival between the groups (*P* = 0.585; Fig. 2). Median OS was 52.0 months (IQR: 42.0–62.0) for early-onset CRC and 50.0 months (IQR: 42.0–59.8) for LOCRC. Mean OS was 50.9 ± 14.9 vs. 48.6 ± 16.1 months, respectively (*P* = 0.128; Table 1). These results indicate comparable long-term outcomes between early-onset CRC and late-onset CRC patients.

*Clinical and pathological features* The difference in tumor location between the two types of CRC was not statistically significant (*P* = 0.901; Table 1). Early-onset CRC patients were more likely to present with advanced stage (stage IV) compared to late-onset CRC patients (24.44% vs. 13.45%, *P* < 0.001; Table 3). Approximately 41.67% and 24.44% of early-onset CRC patients had stage III and IV disease, respectively, compared to 38.99% and 13.45% in the late-onset group (Table 3). Early-onset CRC patients also had higher rates of lymph node metastasis than late-onset CRC patients did (61.11% vs. 48.83%, *P* = 0.003; Table 4). Early-onset CRC patients were more likely to present with poorly differentiated CRC than late-onset CRC patients were (29.81% vs. 12.70%, *P* < 0.001; Table 3). In addition, younger patients also had more mucinous or signet-ring tumors than did the reference group (22.40% vs. 14.17%, *P* = 0.011; Table 3). However, there was no significant difference in perineural invasion or vascular invasion between early-onset and late-onset CRC patients (33.50% vs. 32.53%, *P* = 0.871; Table 3).

**Table 3:** Clinicopathological characteristics of EOCRC and LOCRC patients

	Early-onset CRC (< 50 years, *N* = 180)		Late-onset CRC (≥ 50 years, *N* = 1026)		*P*
Tumor stage	*N*	%	*N*	%	< 0.001
I	25	13.89%	200	19.49%	
II	36	20.00%	288	28.07%	
III	75	41.67%	400	38.99%	
IV	44	24.44%	138	13.45%	< 0.001
Pathological features	*N* = 161		*N* = 953		
Well differentiation	11	6.83%	142	14.90%	0.009
Moderately differentiation	102	63.35%	691	72.40%	0.023
Poor differentiation	48	29.81%	120	12.70%	< 0.001
	*N* = 161		*N* = 953		
Signet ring cell formation or Mucinous adenocarcinoma	36	22.40%	135	14.17%	0.011
	*N* = 161		*N* = 953		
Perineural invasion or vascular invasion	54	33.50%	310	32.53%	0.871
Microsatellite Instability Status	*N* = 164		*N* = 956		< 0.001
pMMR	138	84.10%	896	93.72%	
dMMR	26	15.90%	60	6.28%

**Table 4:** TNM stage distribution in EOCRC and LOCRC patients

	Early-onset CRC		Late-onset CRC		*P*
	*N*	%	*N*	%	
T					
T1	12	6.67%	108	10.53%	0.025
T2	33	18.33%	246	23.98%	
T3	111	61.67%	509	49.61%	
T4	24	13.33%	163	15.89%	
N					
N0	70	38.89%	525	51.17%	0.003
N1 and N2	110	61.11%	501	48.83%	
M					
M0	136	75%	888	86.55%	< 0.001
M1	44	25%	138	13.45%	

*Immunohistochemistry features* For immunohistochemical feature analysis, 86 patients were detected to be dMMR, 26 early-onset and 60 late-onset CRC patients were detected to be dMMR, and some MMR gene deficiencies contained several proteins (Table 5). dMMR tumors were more likely to be present in early-onset CRC patients than in late-onset CRC patients (15.90% vs. 6.28%, *P* < 0.001; Table 3). In early-onset CRC patients, dMMR tumors occurred mostly in the left-sided CRC (83.33% vs. 59.62%, *P* = 0.027; Table 5).

**Table 5:** MMR deficiency features of the EOCRC and LOCRC patient groups

	Early-onset CRC		Late-onset CRC		*P*
	*N*	%	*N*	%	
dMMR					
MLH1 (−)	4	15.38%	3	5.00%	
PMS2 (−)	2	7.69%	3	5.00%	
MSH2 (−)	2	7.69%	4	6.70%	
MSH6 (−)	1	3.85%	4	6.70%	
MLH1 + PMS2 (−)	13	50.00%	33	55.00%	
MSH2 + MSH6 (−)	5	19.23%	13	21.70%	
Tumor location					
Right-sided CRC*	4	16.67%	21	40.38%	0.027
Lift-sided CRC	20	83.33%	31	59.62%	

*The transverse colon is not included

*Features of right- vs. left-sided early-onset CRC* The majority of early-onset CRC cases were left-sided, with tumors most commonly located in the rectum (58.8%) and sigmoid colon (28.4%), followed by the descending colon and splenic flexure (Table 6). Among right-sided early-onset CRC patients, 40.91% were diagnosed at an advanced stage (stage IV), compared to 20.27% of left-sided cases; however, this difference was not statistically significant (*P* = 0.143; Table 6). No significant differences were observed between right- and left-sided early-onset CRC patients in terms of age, sex, BMI, pathological features, or receipt of chemotherapy.

**Table 6:** Various features of different tumor locations in EOCRC patients

	Right-side CRC (*N* = 22)		Lift-side CRC (*N* = 148)		P*	Transverse CRC (*N* = 10)	
	*N*	%	*N*	%		*N*	%
Sex					1		
Female	12	54.55%	78	52.70%		3	30.00%
Male	10	45.45%	70	47.30%		7	70.00%
Age					0.556		
10–19	0		4	2.70%		0	
20–29	0		7	4.70%		0	
30–39	6	27.27%	30	20.30%		3	30.00%
40–49	16	72.73%	107	72.30%		7	70.00%
Tumor location							
Cecum	5	22.73%					
Ascending colon	14	63.64%					
Hepatic flexure	3	13.64%					
Splenic flexure			5	3.40%			
Descending colon			14	9.40%			
Sigmoid colon			42	28.40%			
Rectum			87	58.80%			
Transverse colon						10	
Body mass index (kg/m^2^)					0.234		
Underweight [< 18.5]	2	9.09%	11	7.40%		1	10.00%
Normal weight [18.5–24.9]	18	81.82%	104	70.30%		5	50.00%
Overweight [25.0–29.9]	1	4.55%	31	20.90%		3	30.00%
Obese [≥ 30.0]	1	4.55%	2	1.40%		1	10.00%
Tumor stage					0.143		
I	2	9.09%	23	15.54%		0	
II	5	22.73%	30	20.27%		1	10.00%
III	6	27.27%	65	43.92%		4	40.00%
IV	9	40.91%	30	20.27%		5	50.00%
Pathological features	*N* = 21		*N* = 133		0.604	*N* = 7	
Well differentiation	1	4.76%	10	7.52%		0	
Moderately differentiation	12	57.14%	86	64.66%		4	57.14%
Poor differentiation	8	38.10%	37	27.82%		3	42.86%
	*N* = 21		*N* = 133			*N* = 7	
Signet ring cell formation or Mucinous adenocarcinoma	6	28.57%	28	21.10%	0.115	2	28.57%
	*N* = 21		*N* = 133			*N* = 7	
Perineural invasion and (or) vascular invasion	7	33.33%	44	33.10%	1	3	42.86%

*P values calculated by comparing right-side and left-side CRC groups only using Chi-square or Fisher’s exact test

## Discussion

Recent studies have reported a rising incidence of CRC in younger adults, highlighting the need to better understand early-onset CRC characteristics. This study aimed to comprehensively assess the clinical, pathological, and molecular features of early-onset CRC patients in China and compare them with late-onset CRC cases. Our findings showed that the sex distribution in early-onset CRC patients was nearly balanced, while male predominance was observed in late-onset CRC, consistent with data from a Western Australia population [37]. In contrast, studies from Western countries report a higher proportion of males in early-onset CRC [38, 39]. Regarding tumor location, there was no significant difference between early-onset CRC and late-onset CRC; tumors were similarly distributed across right-sided, left-sided, and rectal locations, aligning with previous findings in China [40]. While U.S. studies have shown a higher rate of mucinous and signet-ring cell histology in right-sided early-onset CRC [31, 36, 41], our data did not confirm this pattern. These findings suggest that early-onset CRC in China may have distinct clinicopathological characteristics compared to Western populations, fulfilling the study’s aim to address regional differences in disease presentation.

In this study, we discovered that early-onset CRC patients had more advanced stage (stage IV) than late-onset CRC patients did. Similar results have been reported in previous studies. In a retrospective study, 61.8% and 46% of patients had advanced stage early-onset CRC and late-onset CRC, respectively [34]. Our research also revealed that patients with early-onset CRC were more likely to present with poorly differentiated CRC and more mucinous or signet-ring tumors than late-onset CRC patients were, which is consistent with previous results [42]. Early-onset CRC patients present with advanced stage, poorly differentiated tumors, and more mucinous or signet-ring tumors than early-onset CRC patients did, which was observed in early-onset CRC patients, indicating unfavorable tumor biology and a worse oncological prognosis. Although early-onset CRC patients are generally diagnosed at a younger age and often present with more aggressive pathological features, our survival analysis revealed no significant difference in overall survival compared to late-onset CRC patients. This finding aligns with several previous studies, suggesting that younger patients may benefit from better baseline health and treatment tolerance, potentially offsetting the adverse impact of aggressive tumor biology [43, 44].

According to our results, dMMR tumors were more common in early-onset CRC than in late-onset CRC, and dMMR tumors were more common in the left-sided colon and rectum in early-onset CRC patients.

Compared with those in late-onset CRC patients, the median and mean durations of symptoms were much longer in early-onset CRC patients. The case‒control study revealed that the median time to treatment from symptom onset in rectal cancer patients was 217 days for patients under the age of 50, whereas it was 29.5 days for individuals above the age of 50 [45]. A lack of knowledge of colorectal cancer-related symptoms in patients with early-onset CRC, a low rate of early screening, and the unwillingness of certain patients to seek medical care may contribute to the delay in diagnosis in early-onset CRC patients. We found that delayed diagnosis was not directly related to advanced stage in early-onset CRC patients. This could instead be explained by a more aggressive biological behavior, as demonstrated by the histological differences. The advanced stage observed in early-onset CRC may reflect a more aggressive tumor biology, as indicated by the higher prevalence of poor differentiation and mucinous or signet-ring histological subtypes in this group. Previous work has demonstrated that delayed diagnosis does not have a negative influence on the stage of the disease at presentation, which could not easily explain why there are present with advanced stage in early-onset CRC patients [35]. Conversely, according to another study, the delay in diagnosis might be one of the reasons for the increased proportion of patients diagnosed with advanced disease [46]. More work needs to be done to reveal the reasons for these two different results.

This study revealed that in early-onset CRC patients, only a few were diagnosed with CRC by colonoscopy screening. In 2018, the American Cancer Society (ACS) changed the recommended age for those at average risk to begin screening from 50 to 45 years [47]. The recommendation is based on disease burden and results from microsimulation modeling studies. In China, the guidelines recommend that individuals over 50 years (50–75) and at average risk undergo CRC screening [48]. Given that only 2.8% of early-onset CRC cases in this study were detected through colonoscopy screening, and the average age at diagnosis was 42 years, these findings support the need to re-evaluate current screening guidelines and consider lowering the starting age for average-risk individuals. However, given China's large population and limited healthcare resources, we should carefully consider whether we need to readjust the starting age of screening for people at average risk. In this study, approximately 45.0% of patients presented with intestinal bleeding as the chief presenting symptom in early-onset CRC patients. In appropriate situations, it may be reasonable to consider colonoscopy in young patients with persistent hematochezia, especially in regions with limited resources. Future research needs to focus on age-stratified characteristics, and a microsimulation model is needed to guide future screening strategies and decrease the disease burden.

In this study, we found that younger patients were more likely to have a family history of CRC. Research has indicated that a family history of CRC is strongly associated with an increased risk of early-onset CRC; approximately 26% of patients with early-onset CRC have a family history of CRC [49]. A family history of CRC is a more important factor associated with early-onset CRC than with late-onset CRC. Assessment of genetic risk on the basis of family history is essential for young patient care.

Given the rising incidence of early-onset CRC, primary prevention strategies are becoming increasingly important. While genetic predisposition accounts for a subset of cases, a growing body of evidence highlights the role of modifiable risk factors. Therefore, enhancing public awareness, promoting healthy lifestyle choices, and considering earlier screening for high-risk individuals may contribute to reducing early-onset CRC burden. Future public health policies should incorporate targeted education and preventive strategies specifically tailored for younger populations.

In conclusion, early-onset CRC has distinct epidemiology, pathophysiology, and molecular characteristics compared with late-onset CRC in China. The preclinical symptoms, family history, and genetic features linked with early-onset CRC should be known to clinicians to aid in the diagnosis of young patients with CRC and to improve their disease prognosis. In the future, large sample sizes and multicenter studies are needed to further understand the clinical features and pathophysiology of patients with early-onset CRC and why there are differences between the two types of CRC.

### Strengths and limitations of this study

This study focused on examining the clinical, pathological, and molecular features of early-onset CRC patients in China, and comprehensively evaluated various characteristics across age groups in relation to CRC outcomes.

This retrospective, single-center study limits causal inference and may affect the generalizability of the findings; future multicenter prospective studies are warranted to validate these results across broader populations.

Due to limited molecular data, BRAF mutation or promoter methylation status were unavailable in most cases with MLH1 loss. Future studies should incorporate comprehensive molecular profiling to clarify the etiology and guide appropriate genetic counseling and surveillance.

## Data Availability

No datasets were generated or analysed during the current study.

## Declarations

### Inclusion of identifiable human data

Generated statement: No potentially identifiable human images or data are presented in this study.
